# CRH neurons in the AM-ACC circuit drive chronic pain and anxiety comorbidity

**DOI:** 10.3389/fnins.2026.1809769

**Published:** 2026-04-10

**Authors:** Qingzan Zhao, Chaofan Lu, Tianen Si, Ziang Li, Xiuhua Ren, Liujie Zhao, Yidan Zhang, Jingjing Zhang, Sen Zhao, Weidong Zang, Jing Cao

**Affiliations:** 1 Department of Anatomy, Basic Medical College, Zhengzhou University, Zhengzhou, Henan, China; 2 School of Medical Sciences, Academy of Chinese Medical Sciences, Henan University of Chinese Medicine, Zhengzhou, Henan, China; 3 Zhengzhou Key Laboratory of Sports Nutrition and Health, School of Sports and Physical Education, Zhengzhou University, Zhengzhou, Henan Province, China; 4 Medical Research Center, Chest Hospital of Zhengzhou University, Zhengzhou, Henan, China; 5 Institute of Neuroscience, Zhengzhou University, Zhengzhou, Henan, China

**Keywords:** anterior cingulate cortex, anteromedial thalamic nucleus, anxiety, corticotropin-releasing hormone, pain

## Abstract

Chronic pain induces anxiety disorders through aberrant interactions between the limbic system and sensory pathways, forming refractory comorbidity. The anterior cingulate cortex (ACC), a key hub for integrating pain and emotion, contains corticotropin-releasing hormone (CRH) neurons closely associated with stress responses. However, the thalamic input mechanisms mediating the pain and anxiety comorbidity remain unclear. Here, through immunofluorescence staining, calcium imaging, neural circuit tracing, patch-clamp recording, and optogenetics- or chemogenetics-based manipulations, we re-veal that the anteromedial thalamic nucleus (AM), as a pivotal nucleus of the anterior thalamic group, specifically regulates ACC^CRH^ neurons via a glutamatergic pathway, forming an AM^CaMKIIɑ^-ACC^CRH^ neural circuit that collectively drives the progression of pain-anxiety comorbidity. This study is to elucidate the central role of the AM^CaMKIIɑ^, ACC^CRH^ neurons, and AM^CaMKIIɑ^-ACC^CRH^ neural circuit in encoding the emotional dimen-sion of pain. It provides a novel theoretical framework for targeting this neural circuit in the treatment of pain-anxiety comorbidity.

## 1 Introduction

Chronic pain, a significant public health challenge affecting over 30% of the global population (Cohen et al., 2021), is inherently a persistent stressor. It can significantly increase the risk of comorbid emotional disorders such as anxiety through complex neurobiological interactions (Vambheim et al., 2021; Yalcin et al., 2014; Zhuo, 2016; Hooten, 2016). The vicious cycle formed between pain perception and negative emotions not only severely compromises patients’ quality of life but also substantially increases the difficulty of clinical treatment (Wan et al., 2025). The traditional view holds that pain information is transmitted via the peripheral sensory nervous system to higher brain centers for perception. However, under chronic pain conditions, aberrant interactions between nociceptive pathways and core affective processing pathways (e.g., the limbic system) are considered a key mechanism leading to emotional dysfunction (Jiang et al., 2025; Chan et al., 2012; Thompson and Neugebauer, 2019). Delving into the neural circuit basis of these interactions is of great importance for developing novel therapeutic strategies targeting emotional disorders comorbid with chronic pain.

As a key hub of the limbic system, the ACC plays a central role in the pathogenesis of chronic pain and its comorbid emotional disorders by integrating affective and cognitive information (Jia et al., 2025; Bliss et al., 2016; Zhuo, 2008). Clear evidence indicates that activating glutamatergic neurons within the ACC can induce pain and anxiety-like behaviors, directly driving the pain-anxiety comorbidity (Gao et al., 2022; Ba et al., 2025; Shen et al., 2020). CRH, also known as corticotropin-releasing factor (CRF), is the activating factor of the hypothalamic–pituitary–adrenal axis and has recently been found to be widely expressed in the cerebral cortex. These neurons play a central regulatory role in the body’s stress response and are closely associated with the development of anxiety disorders (Chaves et al., 2021; Tafet and Nemeroff, 2020). Notably, corticotropin-releasing hormone (CRH) neurons also reside within the ACC (Zhang et al., 2019). However, whether and how ACC^CRH^ neurons participate in the regulation of pain-anxiety comorbidity remains unclear.

The anteromedial thalamic nucleus (AM), a key component of the anterior thalamic nuclei, forms the core connection of the classic Papez circuit via its dense projections to the ACC—a circuit long considered primarily responsible for regulating emotional memory (Aggleton et al., 2022; Kamali et al., 2023). Notably, however, recent studies have revealed abnormalities in white matter integrity within the Papez circuit in patients with anxiety disorders and depression (Li et al., 2023), suggesting its likely involvement in the genesis and development of negative emotions. Although previous research has elucidated the role of the AM in epilepsy propagation and spatial memory consolidation through its connections with the hippocampus and limbic cortex (Moreira-Holguín et al., 2022; de Lima et al., 2017; Szabó et al., 2022; Nelson, 2021; Roy et al., 2022), its function in pain perception and negative emotion regulation has long been overlooked. The principal neuronal type within the AM is glutamatergic neurons (Gonzalo-Ruiz et al., 1996). As a critical relay station transmitting information from the thalamus to the ACC, the AM possesses a unique anatomical advantage: it can simultaneously integrate spatiotemporal contextual information from the hippocampus and nociceptive input signals from brainstem nuclei (Bubb et al., 2017). This characteristic strongly suggests that the AM may act as a pivotal neural hub, converting pain perception information into emotional responses. Nevertheless, specific functional studies on the AM-ACC neural circuit in chronic pain and its associated emotional disorders remain highly scarce.

Therefore, this study aims to investigate whether, in a complete Freund’s adjuvant (CFA)-induced chronic inflammatory pain model, CRH neurons within the ACC mediate pain-related anxiety-like behaviors by receiving and integrating thalamic glutamatergic input signals from the AM. This research will provide new and crucial evidence for elucidating the neural circuit mechanisms underlying the comorbidity of chronic inflammatory pain and anxiety.

## 2 Materials and methods

### 2.1 Animals

Male C57BL/6 mice, aged 6–8 weeks with an initial body weight of 20–25 g, were used. The mice were housed under controlled conditions: temperature 24 ± 1 °C, humidity 45 ± 5%, and a 12-h light/dark cycle (light phase 08:00–20:00). To eliminate cage effects, mice were housed 4–5 per cage. They were provided ad libitum access to feed and water. All mice were acclimatized for at least 7 days prior to experiments. All animal experiments and procedures were conducted in strict compliance with the guidelines of the Animal Care and Use Committee of Zhengzhou University (Ethics Approval No.: ZZUIRB GZR2023-138).

### 2.2 Establishment of chronic inflammatory pain model

Mice were anesthetized by induction with 3% isoflurane and then maintained under 1.5% isoflurane anesthesia. The plantar surface of the left hind paw was disinfected, and 25 μL of Complete Freund’s Adjuvant (CFA, Sigma-Aldrich) was administered via subcutaneous injection into the central region of the plantar surface using an insulin syringe. Control mice received an equal volume of sterile saline (Zhang et al., 2022). Mechanical pain threshold testing was performed on days 1, 3, and 7 post-modeling. Anxiety-like behavioral tests were conducted on days 5 and 6. All procedures were performed at a controlled room temperature of 24 °C to minimize stress-induced interference.

### 2.3 Behavioral tests

Mechanical allodynia testing was conducted in a sound-attenuated behavioral laboratory. Mice were placed in cages (9 × 7 × 10 cm) for a 30-min acclimation period. The plantar center of the left hind paw was vertically stimulated using 0.07 g and 0.4 g Von Frey filaments (North Coast Medical, Inc.), respectively. Each stimulation lasted for 2 s with an inter-stimulus interval of at least 30 s. The number of positive responses (paw withdrawal, licking, or shaking) out of 10 stimulations was recorded, and the paw withdrawal frequency (PWF) was calculated as (number of positive responses/10) × 100% (Su et al., 2021).

The open field test (OFT) utilized a 45 × 45 × 40 cm arena. The floor of the arena was virtually divided into a 4 × 4 grid within the video tracking system, with the central 4 squares defined as the center zone. Initially, the mice were placed in the center of the arena floor facing away from the experimenter, then their activities within 5 min were video-recorded, and the percentage of time spent in the center zone relative to the total time as well as the total distance traveled were analyzed via Smart 3.0 software.

The elevated plus maze (EPM) test was conducted using a plus-shaped maze consisting of two open arms (30 × 5 cm) and two enclosed arms (30 × 5 × 15 cm), elevated 50 cm above the floor. Initially, the mice were placed on the central platform facing an open arm and away from the experimenter, then their activities within 5 min were recorded, and the percentage of time spent in the open arms as well as the total distance traveled were analyzed. All behavioral tests were performed between 09:00 and 16:00. The apparatuses were thoroughly cleaned with 75% ethanol between testing sessions to eliminate residual odors from previous subjects (Yin et al., 2020).

### 2.4 Stereotaxic surgery

Mice were anesthetized by induction with 3% isoflurane and secured in a stereotaxic frame, with anesthesia maintained at 1.5% isoflurane. Erythromycin eye ointment was applied to protect the eyes. The hair on the cranial vertex was shaved, and the scalp was disinfected. A midline sagittal incision was made to expose the bregma and lambda. The bregma was set as the coordinate origin (AP: 0.00 mm, ML: 0.00 mm, DV: 0.00 mm). The mouse was considered to be in a standard stereotaxic position when the lambda and bregma were level within the same *Y*-axis (DV coordinate difference ≤ 0.03 mm), and the DV coordinates at points 2.00 mm lateral to the left and right of the bregma on the skull surface differed by ≤ 0.03 mm. This allowed for targeting brain nuclei according to the brain atlas coordinates. A fine drill bit (0.5 mm) was used to create burr holes at the target regions: ACC at AP +0.38 mm, ML −0.25 mm, DV −1.65 mm (for virus injection)/−1.45 mm (for optical fiber); AM at AP −0.70 mm, ML −0.50 mm, DV −3.65 mm (for virus injection). Viral suspensions were injected using a microinjection pump at a rate of 20 nL/min, with volumes of 200 nL for ACC and 100 nL for AM. After completion of the injection, the needle was left in place for 10 min before slow withdrawal, followed by suturing of the scalp. After the optical fiber implantation procedure was finished, the optical fiber was secured with dental cement. The mice were housed individually for 24 h postoperatively and then returned to group housing (Zhang et al., 2024). All viral injections in this study were performed in the right cerebral hemisphere of mice. Owing to the contralateral innervation of the brain, mechanical pain behaviors were assessed in the hind paw contralateral to the injection side (i.e., the left hind paw) during behavioral testing.

### 2.5 Virus list

**Table 1:** 

Function	Full viral name	Viral titer	Abbreviation
CRH neuron-specific Cre tool virus	rAAV2/9-CRH-CRE-WPRE-hGH polyA	≥2 × 10^12^ vg/ml	AAV-CRH-Cre
CRH neuron reporter virus (EGFP)	rAAV2/9-CRH-EGFP-WPRE-hGH polyA	≥2 × 10^12^ vg/ml	AAV-CRH-EGFP
Cre-dependent chemogenetic activation virus	rAAV2/9-EF1a-DIO-hM3D(Gq)-EGFP-WPREs	≥2 × 10^12^ vg/ml	AAV-DIO-hM3D(Gq)-EGFP
Cre-dependent chemogenetic inhibition virus	rAAV2/9-EF1a-DIO-hM4D(Gi)-EGFP-WPREs	≥2 × 10^12^ vg/ml	AAV-DIO-hM4D(Gi)-EGFP
Cre-dependent control virus	rAAV2/9-EF1a-DIO-EGFP-WPRE-hGH polyA	≥2 × 10^12^ vg/ml	AAV-DIO-EGFP
Cre-dependent calcium indicator virus	rAAV2/9-EF1α-DIO-GCaMp6s-WPRE-hGH polyA	≥2 × 10^12^ vg/ml	AAV-DIO-GCaMp6S
CaMKIIa neuron-specific Cre tool virus	rAAV2/9-CaMKIIa-CRE-WPRE-hGH polyA	≥2 × 10^12^ vg/ml	AAV-CaMKIIa-Cre
Chemogenetic activation virus for CaMKIIα neurons	rAAV2/9-CaMKIIa-hM3D(Gq)-EGFP-WPRE-hGH polyA	≥2 × 10^12^ vg/ml	AAV-CaMKIIa-hM3D(Gq)-EGFP
Chemogenetic inhibition virus for CaMKIIα neurons	rAAV2/9-CaMKIIa-hM4D(Gi)-EGFP-WPRE-hGH polyA	≥2 × 10^12^ vg/ml	AAV-CaMKIIa-hM4D(Gi)-EGFP
Control virus for CaMKIIα neurons	rAAV2/9-CaMKIIa-EGFP -WPRE-hGH polyA	≥2 × 10^12^ vg/ml	AAV-CaMKIIa-EGFP
Optogenetic activation virus for CaMKIIα neurons	rAAV2/9-CaMKIIa-hChR2(H134R)-mCherry-WPRE-hGH polyA	≥2 × 10^12^ vg/ml	AAV-CaMKIIa-ChR2-mCherry
Optogenetic activation virus for CaMKIIα neurons	rAAV2/9-CaMKIIa-hChR2(H134R)-EGFP-WPRE-hGH polyA	≥2 × 10^12^ vg/ml	AAV-CaMKIIa-ChR2-EGFP
Optogenetic inhibition virus for CaMKIIα neurons	AAV2/9-CaMKIIα-eNPHR3.0-EYFP-WPRE-hGH pA	≥2 × 10^12^ vg/ml	AAV-CaMKIIα-eNPHR-EYFP
Neuronal ablation virus for CaMKIIα neurons	rAAV2/9-CaMKIIa-taCasp3-T2A-TEVp-WPRE-hGH polyA	≥2 × 10^12^ vg/ml	AAV-CaMKIIa-taCasp3
Anterograde transsynaptic Cre tool virus	rAAV2/1-hSyn-CRE-WPRE-hGH polyA	≥1 × 10^13^ vg/ml	AAV2/1-Cre

All adeno-associated viruses were purchased from BrainVTA Inc., Wuhan, China.

### 2.6 Optogenetic/chemogenetic manipulation

Chemogenetic group: A mixture of AAV-CRH-Cre and AAV -DIO-hM3D(Gq)/hM4D(Gi)-EGFP (200 nL), or the control virus AAV-DIO-EGFP, were injected into the ACC; AAV-CaMKIIa-hM3D(Gq)/hM4D(Gi)-EGFP (100 nL), or the control virus AAV-CaMKIIa-EGFP, were injected into the AM. Optogenetic group: AAV-CaMKIIa-ChR2-mCherry, AAV-CaMKIIα-eNPHR-EYFP, or the control virus AAV-CaMKIIa-mCherry/EGFP were injected into the AM at a single site; an optical fiber cannula was implanted above the ACC. Neuronal ablation group: AAV-CaMKIIa-taCasp3, or the control virus AAV-CaMKIIa-Cre, was injected into the AM. Mice were housed for 4 weeks following chemogenetic virus injection and 6 weeks following optogenetic virus injection to allow for stable transgene expression, after which subsequent experiments were performed.

For the chemogenetic group, clozapine N-oxide (CNO) was administered to mice via intraperitoneal (i.p.) injection at a dose of 1 mg/kg, and behavioral tests were conducted 30 min post-administration (Zorrilla et al., 2002).

The optogenetic group received light stimulation via optical fibers with core diameters of 200 μm (Inper Inc., Hangzhou, China). Using a Plexon optogenetic device, 465 nm blue light (20 Hz, 10 ms pulse width, 5 mW power) was used to activate ChR2, and 590 nm yellow light (20 Hz, 10 ms pulse width, 5 mW power) to inhibit eNpHR3.0. To mimic the prolonged duration required for chronic pain to induce anxiety-like behaviors, 2 h of light irradiation were administered daily during behavioral tests to achieve long-term regulation of neurons. To prevent brain damage caused by prolonged laser irradiation, a cyclic stimulation mode of 2 min on/2 min off was adopted.

### 2.7 Tissue processing and sectioning

Mice were deeply anesthetized, and a thoracotomy was performed to expose the heart. Pre-cooled saline (50 mL) was perfused through the left ventricle to clear the blood, followed by perfusion with 30 mL of 4% paraformaldehyde (PFA). The brains were then removed and post-fixed in 4% PFA at 4 °C for 2 h. Subsequently, the brains were sequentially dehydrated in 20 and 30% sucrose solutions (at 4 °C) for cryoprotection. The brains were embedded in OCT compound, and 30 μm-thick coronal sections were prepared using a cryostat. The sections were collected for subsequent immunofluorescence staining or imaging under a fluorescence microscope (Zhang et al., 2024).

### 2.8 Immunofluorescence staining

For single-label staining, brain tissue sections were blocked with PBS containing 0.3% Triton X-100 and 10% goat serum for 2 h, then incubated overnight at 4 °C with primary antibodies: rabbit anti-CRH (Bioss, Cat. No. bs-0246R, 1:100) and/or rabbit anti-c-Fos (Abcam, Cat. No. ab190289, 1:1000). After 3 PBS washes, sections were incubated with secondary antibodies (Cy3-conjugated goat anti-rabbit IgG, Jackson ImmunoResearch, Cat. No. 111-165-003, 1:200; or Alexa Fluor 488-conjugated goat anti-mouse IgG, Jackson ImmunoResearch, Cat. No. 115-545-003, 1:200) at room temperature in the dark for 2 h, then mounted with DAPI-containing anti-fade medium.

For double-label staining (both antibodies are rabbit-derived), a multi-label fluorescence kit (Aifang biological, Cat. No. AFIHC024) was used as follows: Sections underwent microwave antigen retrieval in pH 6.0 citrate buffer, cooled, and washed 3 times with PBS. After blocking with the kit’s peroxidase blocking solution (15 min, room temperature, dark) and 3 PBS washes, sections were blocked with goat serum at 37 °C for 30 min. Rabbit anti-CRH (1,100) was added, incubated overnight at 4 °C in a dark humid chamber, washed 3 times with PBS, then incubated with the kit’s Polymer-HRP goat anti-rabbit secondary antibody (30 min, room temperature, dark) followed by 3 PBS washes. TYR-570 red fluorescent dye was added for 10 min (room temperature, dark), sections were washed 3 times with PBS, then eluted twice with preheated antibody elution buffer (Cat. No. AFIHC038, 37 °C, 15 min each) and washed 3 times with PBS. After re-blocking for 30 min, rabbit anti-c-Fos (1,1,000) was added, incubated overnight at 4 °C, washed 3 times with PBS, and incubated with the kit’s secondary antibody (30 min, room temperature, dark) followed by 3 PBS washes. TYR-520 green fluorescent dye (ready-to-use) was added for 10 min (room temperature, dark), sections were washed 3 times with PBS, spin-dried, and mounted with DAPI-containing anti-fade medium (Zhang et al., 2024). Images were acquired with a Nikon A1 MP^+^ laser scanning confocal microscope. CRH^+^/c-Fos^+^ double-positive cell density was quantified using ImageJ software. The scale bar in all figures is 100 μm.

### 2.9 Vitro calcium imaging

For *in vitro* calcium imaging, mice were injected with AAV-CRH-Cre and AAV-DIO-GCaMp6S viruses in the ACC and AAV-CaMKIIa-GCaMP6s viruse in the AM. After 28 days, the CFA model was established. On day 6 of the model, the mice were decapitated, and the brains were rapidly removed and placed in oxygenated (95% O_2_/5% CO_2_) slicing solution. Coronal slices (300 μm thick) were prepared using a vibratome. Slices were recovered in artificial cerebrospinal fluid (ACSF: 126 mM NaCl, 2.5 mM KCl, 1.25 mM NaH_2_PO_4_, 26 mM NaHCO_3_, 2 mM CaCl_2_, 2 mM MgSO_4_, 10 mM glucose) at 34 °C for 1 h. Calcium signals in response to glutamate stimulation (100 μM) were recorded using a two-photon microscope with a 20 × water immersion objective. Fluorescence signals were analyzed using NIS-Elements software. The change in fluorescence was expressed as Δ*F*/*F*_0_, where *F*_0_ represents the baseline fluorescence. The area under the curve (AUC) and peak amplitude of the calcium transients were analyzed.

### 2.10 Patch-clamp recording

Mice were decapitated, and the brains were rapidly removed and placed in oxygenated (95% O_2_/5% CO_2_) slicing solution. Coronal slices (300 μm thick) were prepared using a vibratome. The slices were recovered in artificial cerebrospinal fluid (ACSF: 126 mM NaCl, 2.5 mM KCl, 1.25 mM NaH_2_PO_4_, 26 mM NaHCO_3_, 2 mM CaCl_2_, 2 mM MgSO_4_, 10 mM glucose) at 34 °C for 1 h. Brain slices containing ACC were secured in a recording chamber and continuously perfused with oxygenated ACSF at a rate of 2 mL/min. EGFP-labeled CRH neurons were identified under an infrared differential interference contrast (IR-DIC) microscope. Borosilicate glass microelectrodes (resistance: 4–6 MΩ) were filled with an internal solution containing 130 mM K-gluconate, 5 mM NaCl, 0.3 mM EGTA, 10 mM HEPES, 2 mM Mg-ATP, and 0.3 mM Na-GTP (pH adjusted to 7.3 with KOH). Whole-cell patch-clamp recordings were performed using a Multiclamp 700B amplifier. In current-clamp mode, step current injections (0–140 pA, 10 pA increments, 200 ms duration) were applied to evoke neuronal responses. The signals were acquired at a sampling rate of 20 kHz. The resting membrane potential (RMP), action potential threshold, firing frequency, and input resistance were recorded and analyzed offline using pClamp 10 software (Zhang et al., 2021).

### 2.11 Neural circuit tracing

Anterograde tracing: AAV-CaMKIIa-ChR2-mCherry was injected into the AM. After 4 weeks, brains were collected, and coronal sections were prepared. Green fluorescent fibers in the ACC were observed under a fluorescence microscope. Retrograde tracing: Fluoro-Gold (FG, Fluorochrome) was injected into the ACC. After 7 days, mice were perfused, and their brains were collected. Sections were examined to identify retrogradely labeled neurons in the AM (Li et al., 2025). Virus co-tracing: the anterograde transsynaptic virus was injected into the AM, while a mixture of AAV-CRH-EGFP (as shown in Supplementary Figure S1A, EGFP highly colocalizes with CRH protein, confirming the excellent targeting efficiency of the virus) and AAV-DIO-mCherry was injected into the ACC.

### 2.12 Statistical analysis

Data are presented as mean ± standard deviation (Mean ± SD), with n representing the number of replicate samples. Statistical analysis was performed using GraphPad Prism 9 software. For comparisons between two independent groups, the unpaired *t*-test, Welch’s *t*-test, or Mann–Whitney *U* test was used according to the results of normality and homogeneity of variance tests. For multiple-group comparisons of repeated-measures data, one-way or two-way analysis of variance (ANOVA) was applied. The normality of residuals was verified, and homogeneity of variance was assessed using the Brown–Forsythe test. Multiple comparisons were corrected with the Bonferroni method. The significance level was set at *p* < 0.05.

The specific statistical tests used for each comparison are provided in the Supplementary Table S1.

### 2.13 Generative AI and AI-assisted technologies

During the preparation of this study, the authors used DeepSeek to enhance the writing fluency and logical coherence of the article. After using this tool, the authors have reviewed and edited the content as needed and take full responsibility for the entire content of the published article.

## 3 Results

### 3.1 CFA model induces inflammatory pain and anxiety-like behaviors in mice

**Figure 1:**
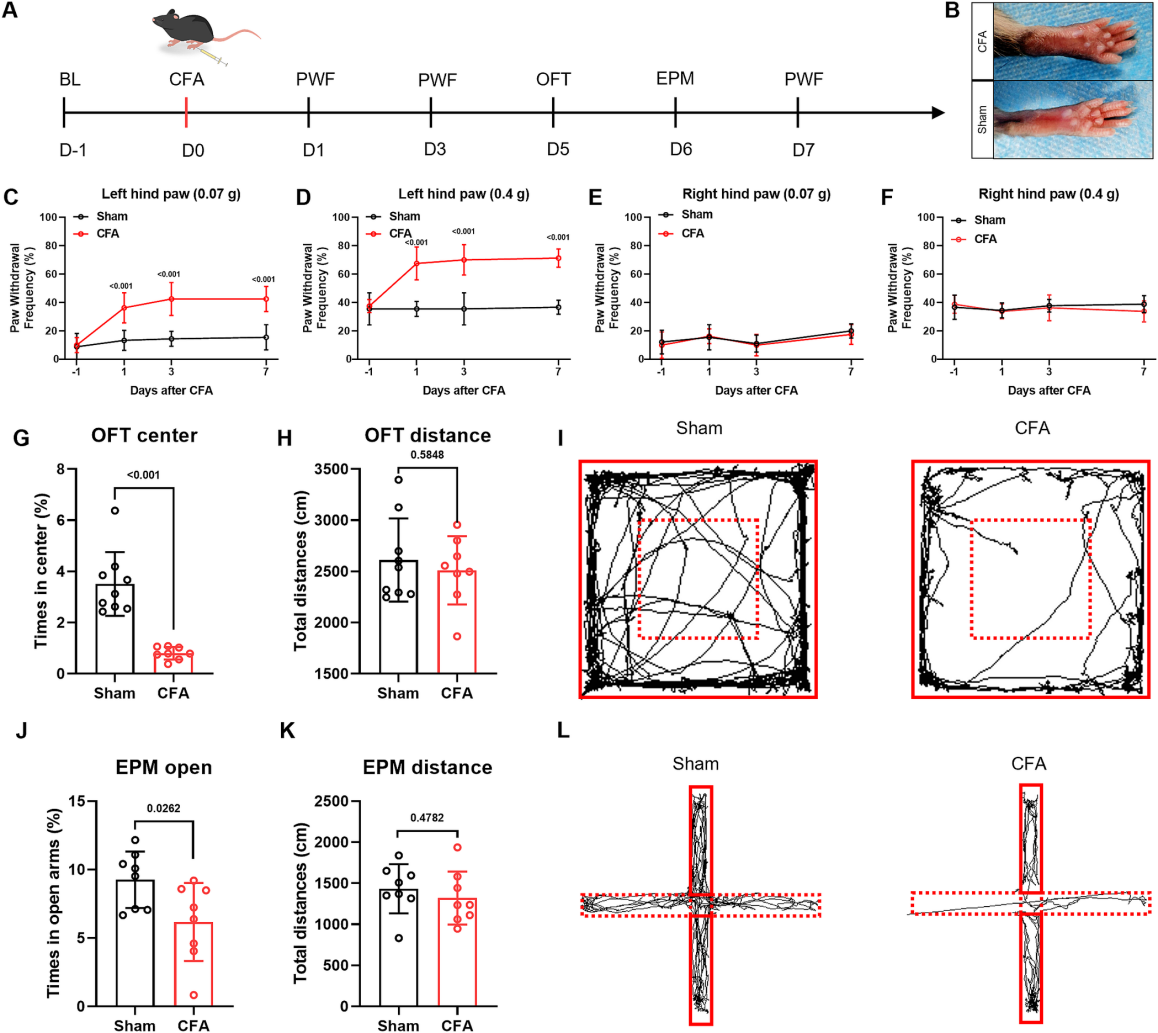
The CFA model induces inflammatory pain and anxiety-like behaviors in mice. **(A)** Experimental design flowchart: Baseline mechanical paw withdrawal frequency (PWF) was measured 1 day prior to CFA injection. On day 0, CFA was injected into the left hind paw. PWFs for both hind paws were assessed on days 1, 3, and 7 post-injection. The open field test (OFT) and elevated plus maze (EPM) test were conducted on days 5 and 6, respectively. **(B)** Representative image showing significant redness and swelling in the CFA-injected paw. **(C–F)** The CFA group showed a significantly increased PWF in response to 0.07 g and 0.4 g von Frey filaments on the injected side on days 1, 3, and 7 post-injection. **(G–L)** On day 5, the OFT revealed a significantly reduced percentage of time spent in the center zone. On day 6, the EPM test showed a significantly reduced percentage of time spent in the open arms. No significant difference was observed in the total distance traveled.

To establish a reliable model for studying pain-anxiety comorbidity, we first induced chronic inflammatory pain by subcutaneously injecting 25 μL of CFA into the left hind paw of C57 mice. Compared to the saline control group, mice in the CFA-injected group exhibited significant redness and swelling in the left hind paw (Figure 1B). Mechanical allodynia testing was performed on days 1, 3, and 7 post-injection (Figure 1A). The CFA group showed a significantly increased paw withdrawal frequency in response to 0.07 g and 0.4 g Von Frey filaments on the left side (*p* < 0.05), while no significant change was observed in the response of the right hind paw (Figures 1C–F). On day 5 post-injection, the open field test revealed that mice in the CFA group spent a significantly lower percentage of time in the center zone (Figures 1G,I). On day 6, the elevated plus maze test showed a significant reduction in the percentage of time spent in the open arms (Figures 1J,L). The total distance traveled did not differ significantly between the two groups (Figures 1H,K), indicating that motor function was unaffected. These results confirm that CFA successfully induced a comorbid phenotype of inflammatory pain hypersensitivity and anxiety-like behaviors.

### 3.2 CRH neuron activity in the ACC is enhanced in CFA model mice

**Figure 2:**
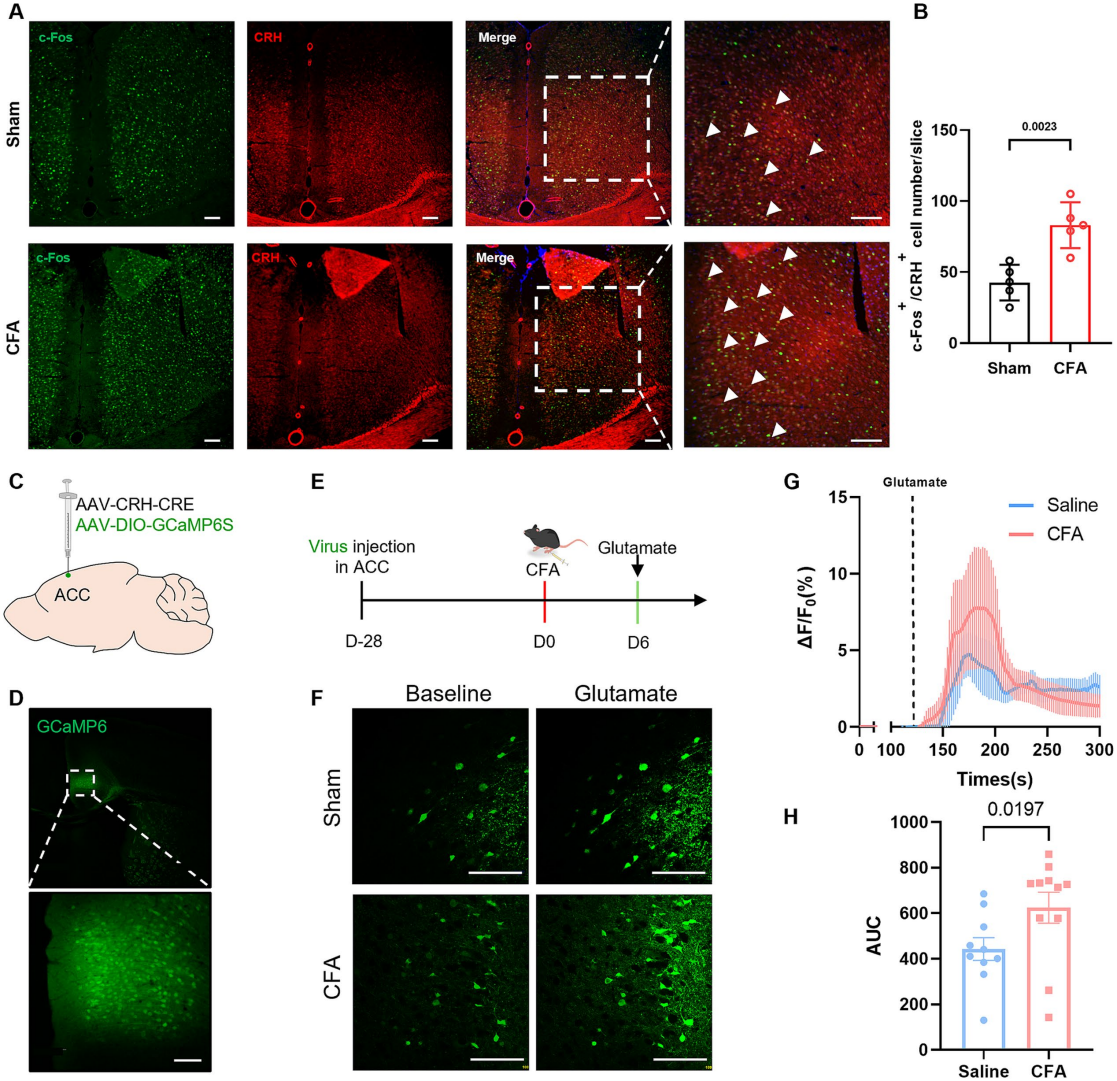
CRH neuron activity in the ACC is enhanced in CFA model mice. **(A, B)** Compared with the Sham group, the co-localization rate of CRH and c-Fos in the ACC of mice in the CFA group was significantly increased. **(C)** Schematic diagram of viral injection in the ACC region. **(D)** Fluorescence image showing viral injection. **(E)** Experimental flowchart. **(F)** Representative fluorescence images of baseline and glutamate-evoked calcium signals in the Sham and CFA groups. **(G)** Calcium signal Δ*F*/*F*_0_ traces comparing Sham and CFA groups. **(H)** Statistical analysis of the area under the Δ*F*/*F*_0_ curve.

Given the pivotal role of the ACC in integrating pain and emotion and the crucial involvement of CRH neurons in stress responses, we next investigated the activity state of CRH neurons within the ACC. Brain tissues were collected for analysis on day 6 post-CFA modeling. Immunofluorescence double staining confirmed that the number of CRH^+^/c-Fos^+^ double-labeled neurons in both the superficial and deep layers of the ACC increased by 1.95-fold in the CFA group compared to the control group (Figures 2A,B). To validate these findings at the functional level, we injected AAV-CRH-Cre and AAV-DIO-GCaMp6S viruses into the ACC (Figures 2C,D). *In vitro* calcium imaging showed that 1 day after CFA modeling, CRH neurons exhibited a greater response to glutamate (Figures 2E,F), and the area under the Δ*F*/*F*_0_ curve of CRH neurons was increased by 1.40-fold (Figures 2G,H). This multi-dimensional evidence consistently indicates significant activation of CRH neurons in the ACC of CFA model mice.

### 3.3 Bidirectional chemogenetic manipulation of CRH neuron activity in the ACC reversibly modulates pain sensitivity and anxiety-like behaviors

To elucidate the functional role of CRH neurons in the ACC in pain-anxiety comorbidity, we employed chemogenetic approaches to bidirectionally modulate their activity. We used CRH promoter-driven Cre virus to label CRH neurons, combined with Cre-dependent DIO virus to achieve precise targeted regulation of CRH neurons.

**Figure 3:**
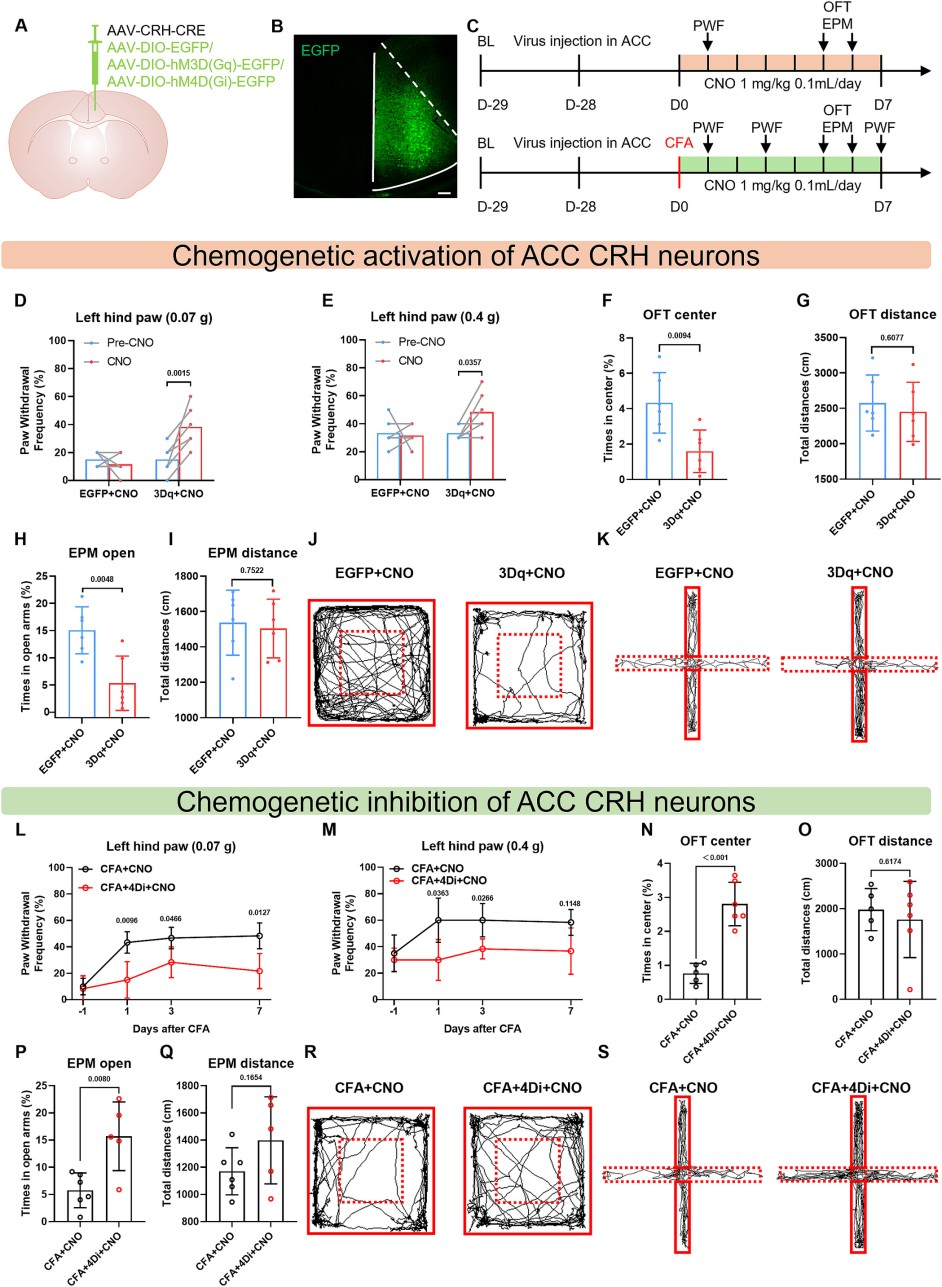
Bidirectional chemogenetic manipulation of ACC^CRH^ neurons reversibly modulates pain and anxiety-like behaviors. **(A)** Schematic diagram of the viral injection site. **(B)** Fluorescence image showing viral expression in the ACC region. **(C)** Behavioral testing flowchart. **(D, E)** After CNO administration, mice showed a significantly increased paw withdrawal frequency in response to mechanical stimulation on the left hind paw. **(F, G)** The hM3Dq + CNO group spent less time in the center zone of the open field test, with no significant difference in total distance traveled. **(H, I)** The hM3Dq + CNO group spent less time in the open arms of the elevated plus maze, with no significant difference in total distance traveled. **(J)** Representative movement trajectories of mice in the open field test. **(K)** Representative movement trajectories of mice in the elevated plus maze test. **(L, M)** Compared to the CFA + CNO group, the CFA + hM4Di + CNO group showed a significantly reduced mechanical paw withdrawal frequency on days 1, 3, and 7 post-CFA injection. **(N, O)** The CFA + hM4Di + CNO group spent more time in the center zone of the open field test, with no significant difference in total distance traveled. **(P, Q)** The CFA + hM4Di + CNO group spent more time in the open arms of the elevated plus maze, with no significant difference in total distance traveled. **(R)** Representative movement trajectories of mice in the open field test. **(S)** Representative movement trajectories of mice in the elevated plus maze test.

First, we specifically activated ACC^CRH^ neurons: the experimental group (3Dq + CNO) were injected with AAV-CRH-Cre and AAV-DIO-hM3D(Gq)-EGFP, the control group (EGFP+CNO) were injected with AAV-CRH-Cre and AAV-DIO-EGFP (Figures 3A,B). The virus-labeled green fluorescent neurons showed excellent colocalization with the red fluorescent neurons labeled with CRH antibody (Supplementary Figure S1B), demonstrating the good targeting specificity of the CRH promoter-driven virus. After sufficient viral expression, mice were intraperitoneally injected with clozapine-N-oxide (CNO, 1 mg/kg/day) for 6 consecutive days to achieve long-term activation of CRH neurons in the ACC and mimic the long-term disease course of the CFA model (Figure 3C). Experimental results showed that the 3Dq + CNO group after CNO activation exhibited a significantly higher paw withdrawal frequency in response to 0.07 g and 0.4 g Von Frey filaments on the left hind paw compared to the 3Dq + CNO group before CNO activation on day 1 (Figures 3D,E). Anxiety-like behavioral tests revealed that the activation group spent a significantly lower percentage of time in the center zone in the open field test on day 5 (Figures 3F,J) and a significantly lower percentage of time in the open arms in the elevated plus maze test on day 6 (Figures 3H,K). The total distance traveled did not differ between the two groups (Figures 3G,I). These results indicate that specific activation of ACC^CRH^ neurons can induce mechanical hyperalgesia and anxiety-like behaviors.

Second, to verify the necessity of ACC^CRH^ neurons in CFA-induced pain and anxiety, we specifically inhibited ACC^CRH^ neurons: the inhibition group (CFA + 4Di + CNO) were injected with AAV-CRH-Cre and AAV-DIO-hM4D(Gi)-EGFP; the control group (CFA + CNO) were injected with AAV-CRH-Cre and AAV-DIO-EGFP (Figure 3A). Mice received intraperitoneal injections of CNO (1 mg/kg/day) for 7 consecutive days post-CFA modeling, and behavioral tests were conducted. Results showed that on days 1, 3, and 7 post-CFA (Figure 3C), the inhibition group exhibited a significantly lower paw withdrawal frequency in response to 0.07 g and 0.4 g Von Frey filaments on the left hind paw compared to the control group (Figures 3L,M). Concurrently, the inhibition group spent a significantly higher percentage of time in the center zone in the open field test and a significantly higher percentage of time in the open arms in the elevated plus maze test (Figures 3N,P). The total distance traveled showed no statistical difference between the two groups (Figures 3O,Q). Representative trajectory diagrams of the OFT and EPM are shown (Figures 3R,S). These findings demonstrate that inhibition of ACC^CRH^ neurons can alleviate CFA-induced mechanical hyperalgesia and anxiety-like behaviors.

### 3.4 AM^CaMKIIα^ neurons innervate and enhance the excitability of ACC^CRH^ neurons

**Figure 4:**
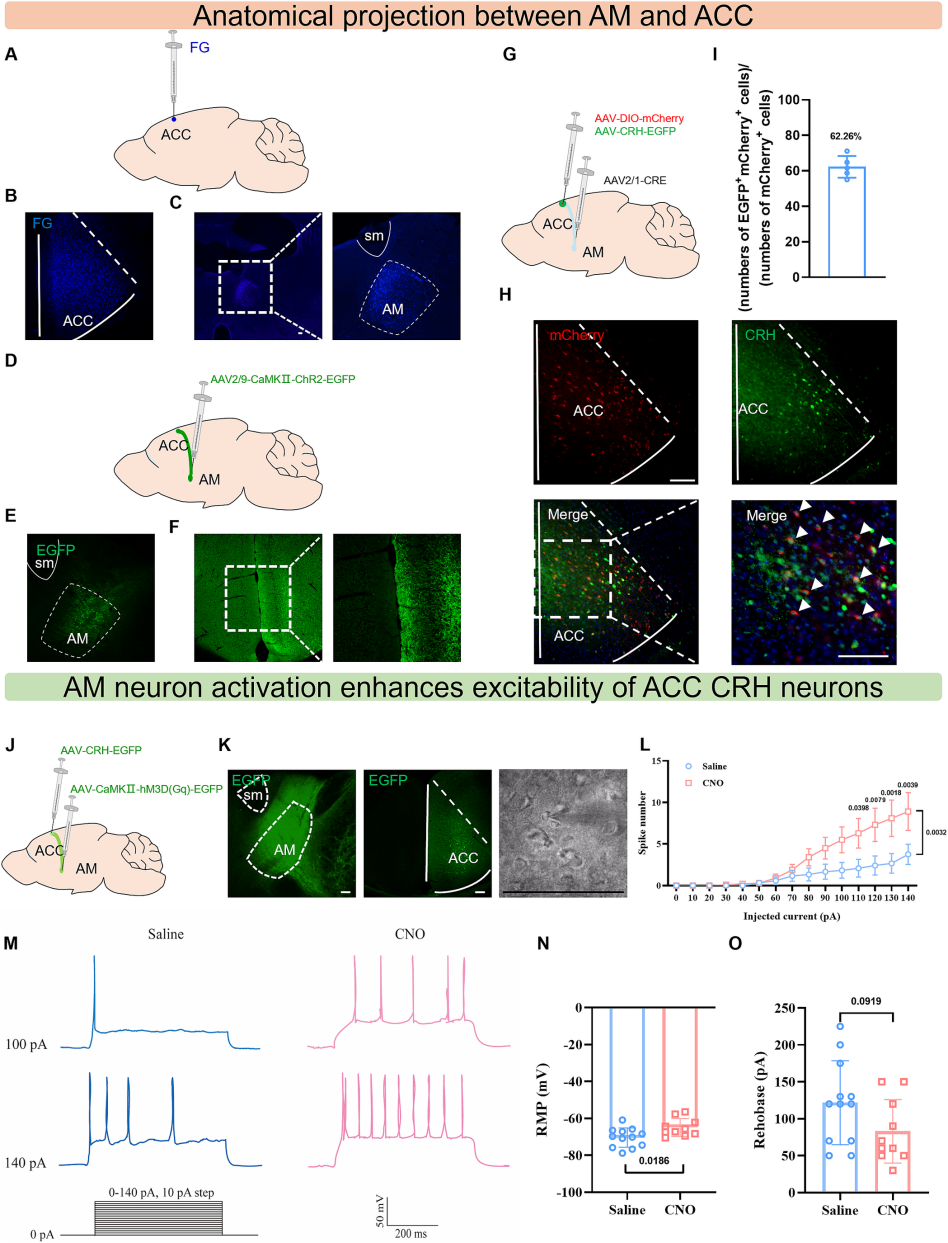
AM^CaMKIIα^ neurons innervate ACC^CRH^ neurons and enhance their synaptic excitability. **(A–C)** Schematic diagram of fluoro gold injection in the ACC region, fluorescence image, and blue somatic fluorescence in the AM region. **(D–F)** Schematic diagram of anterograde tracer virus injection in the AM region, fluorescence image, and green fiber fluorescence in the ACC region. **(G–I)** Schematic diagram of anterograde transsynaptic virus Cre injection in the AM region and Cre-dependent virus plus CRH promoter virus injection in the ACC region, fluorescence image, and co-labeling statistical graph. **(J)** Schematic diagram of chemogenetic activation virus injection in the AM and CRH neuron reporter virus injection in the ACC. **(K)** Representative fluorescence images of viral injection sites in the AM and ACC and infrared image for whole-cell patch-clamp recording. **(L, M)** The CNO perfusion group showed a significantly increased action potential firing frequency in ACC^CRH^ neurons evoked by 110–140 pA current stimulation. **(N)** Compared to the saline group, the CNO perfusion group exhibited a significantly increased resting membrane potential. **(O)** Compared to the saline group, the CNO perfusion group showed a decreasing trend in rheobase current but without statistical significance.

To trace the upstream brain regions of the ACC^CRH^ neurons, we performed multi-level neural tracing verification. First, following injections of the retrograde tracers Fluoro-Gold into the ACC, a substantial number of labeled neurons were observed in the AM (Figures 4A–C). Furthermore, after injection of the optogenetic virus AAV-CaMKIIa-ChR2-EGFP (which can be used as an anterograde tracer virus) into the AM region, dense EGFP^+^ axonal terminals were detected in the ipsilateral ACC (Figures 4D–F). The principal neuronal type within the AM is glutamatergic neurons (Gonzalo-Ruiz et al., 1996), so the above evidence demonstrates that AM glutamatergic neurons form structural connections with the ACC. To further determine whether the AM specifically targets ACC^CRH^ neurons, we injected the anterograde transsynaptic virus AAV2/1-Cre into the AM region, while co-injecting the CRH neuron reporter virus (AAV-CRH-EGFP, the virus-labeled green fluorescent neurons showed good colocalization with CRH antibody-labeled neurons (Supplementary Figure S1A) and a Cre-dependent reporter virus (AAV2/9-DIO-mCherry) into the ACC. Results showed that 62.26% of ACC neurons receiving direct input from the AM (mCherry^+^) were co-labeled with CRH^+^ neurons (Figures 4G–I), providing evidence that AM glutamatergic inputs innervate a subset of ACC^CRH^ neurons.

To dissect the direct functional regulation of AM on ACC^CRH^ neurons, we employed chemogenetic intervention combined with patch-clamp recording *in vitro*. The AM region was injected with AAV-CaMKIIa-hM3D(Gq)-EGFP virus to specifically manipulate glutamatergic neurons, and CRH neurons in the ACC were labeled with AAV-CRH-EGFP. Mice were intraperitoneally injected with 5 mg/kg CNO. 30 min later, the mice were sacrificed to obtain brain tissue sections containing the ACC (Figures 4J,K). Patch-clamp recordings were performed within 3 h: the evoked firing frequency of ACC^CRH^ neurons was significantly increased (Figures 4L,M); the resting membrane potential was depolarized (Figure 4N); and the action potential threshold showed a trend toward reduction (Figure 4O). These electrophysiological evidences indicate that AM glutamatergic inputs drive the hyperactivation state of ACC^CRH^ neurons by directly enhancing their membrane excitability (depolarization) and synaptic output efficacy (increased firing frequency).

### 3.5 Hyperactivation of AM neurons in the CFA inflammatory pain model

**Figure 5:**
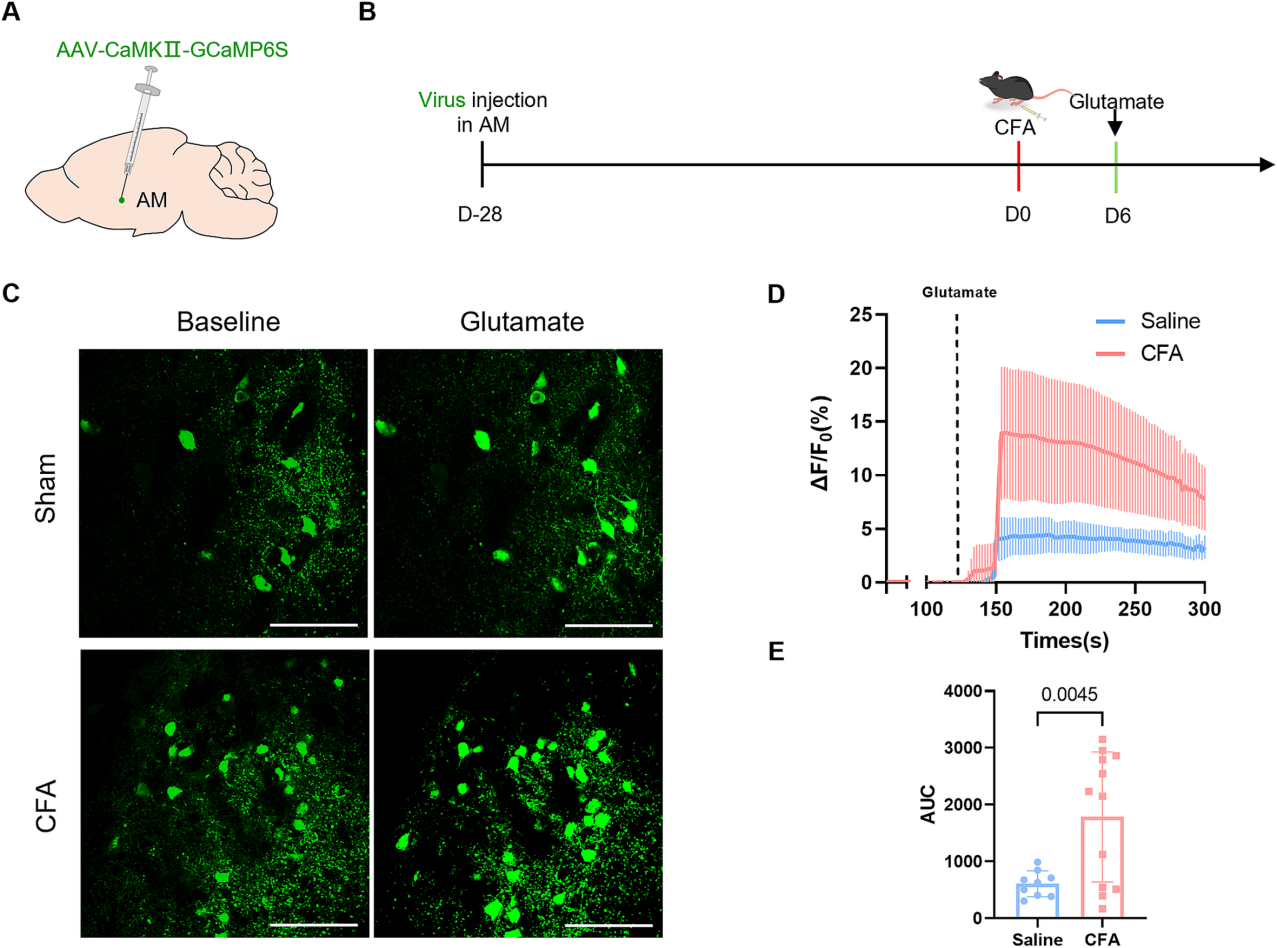
Increased excitability of AM neurons in the CFA model. **(A)** Schematic diagram of the viral injection site. **(B)** On the 6th day after CFA modeling, the mice were sacrificed, and brain slice tissues were harvested for *in vitro* calcium imaging recording. **(C)** Experimental flowchart for *in vitro* calcium imaging and representative fluorescence images of baseline and glutamate-evoked calcium signals in the Sham and CFA groups. **(D)** Calcium signal Δ*F*/*F*_0_ traces comparing the Sham and CFA groups. **(E)** Statistical analysis of the area under the Δ*F*/*F*_0_ curve.

To evaluate the impact of chronic inflammatory pain on the excitability of AM neurons, combining virus-mediated cell-type-specific (AAV-CaMKIIa-GCaMP6s) calcium imaging in vitro, we found that AM glutamatergic neurons in brain slices of the CFA group exhibited significant excitability abnormalities (Figures 5A,B): in two-photon calcium imaging experiments, glutamate perfusion elicited calcium transients with a higher amplitude (Δ*F*/*F*_0_) in the CFA group compared to the Sham group (Figures 5C–E). This evidence indicates that AM glutamatergic neurons become hyperexcitable under the pathological state of CFA-induced chronic pain.

### 3.6 Specific ablation of AM^CaMKIIα^ neurons reverses the pain-anxiety comorbidity phenotype in CFA mice

**Figure 6:**
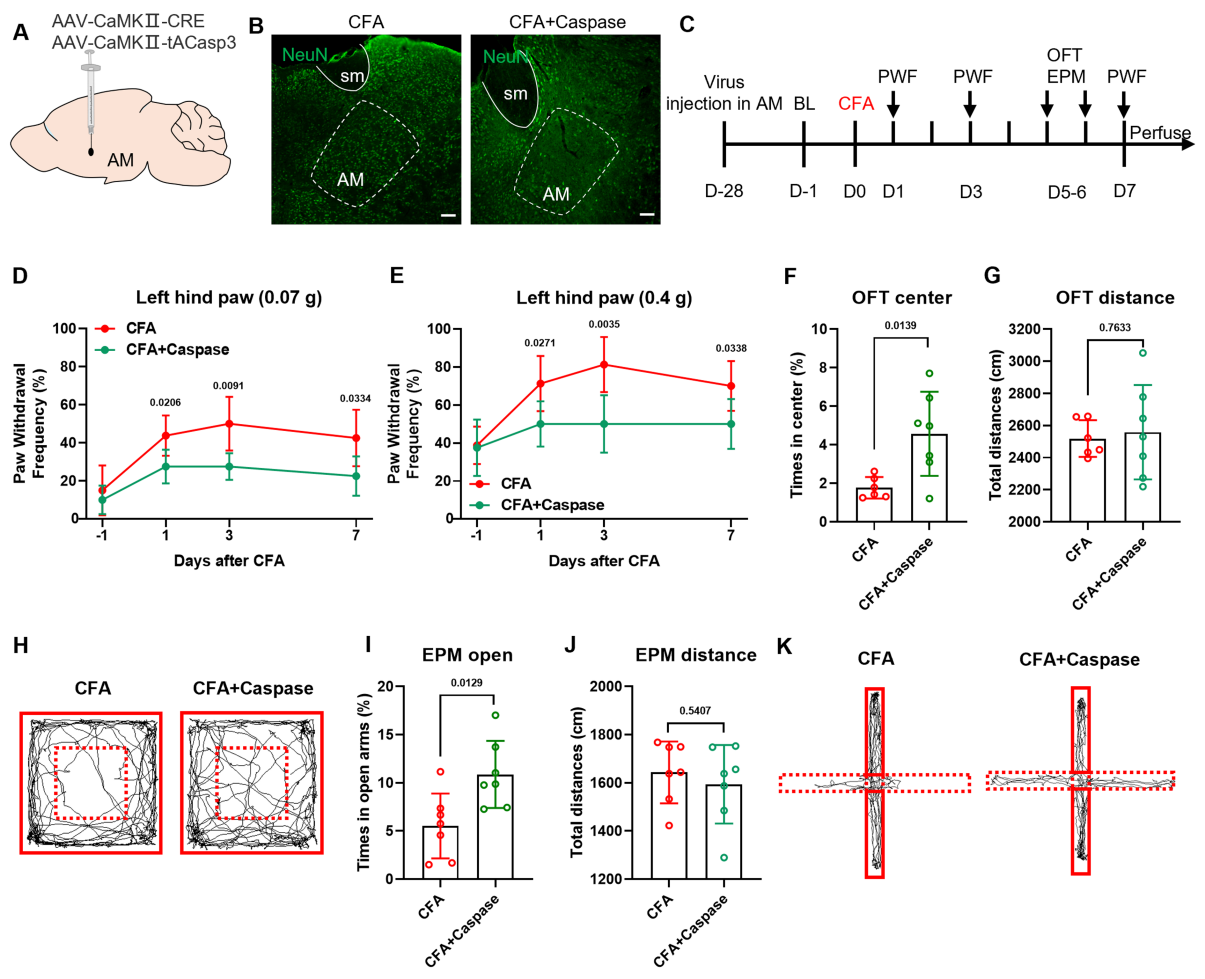
Specific ablation of AM glutamatergic neurons reverses the pain-anxiety comorbidity phenotype in CFA mice. **(A)** Schematic diagram of the viral injection site. **(B)** NeuN fluorescence staining showing viral injection in the AM region. **(C)** Behavioral testing flowchart. **(D, E)** Compared to the CFA group, the CFA + Caspase group showed a significantly reduced mechanical paw withdrawal frequency on days 1, 3, and 7 post-CFA injection. **(F, G)** The CFA + Caspase group spent more time in the center zone of the open field test, with no significant difference in total distance traveled. **(H)** Representative movement trajectories of mice in the open field test. **(I, J)** The CFA + Caspase group spent more time in the open arms of the elevated plus maze, with no significant difference in total distance traveled. **(K)** Representative movement trajectories of mice in the elevated plus maze test.

To verify the causal role of CaMKIIα-expressing neurons in the AM in pain-emotion comorbidity, we injected AAV-CaMKIIa-taCasp3 virus into the right AM region to achieve specific ablation of glutamatergic neurons. After 28 days, allowing sufficient time for viral expression and ablation, chronic inflammatory pain was induced by injecting CFA into the left hind paw (Figures 6A–C). Behavioral analysis revealed that compared to the CFA control group, the CFA + Caspase group showed a significantly reduced paw withdrawal frequency in response to mechanical stimulation with 0.07 g and 0.4 g Von Frey filaments (Figures 6D,E). Further anxiety-like behavior tests demonstrated that neuronal ablation significantly increased the time spent exploring the center zone in the open field test (Figures 6F,H) and the time spent in the open arms of the elevated plus maze (Figures 6I,K). No significant differences were observed in the total distance traveled in either test (Figure 6G,J). These data confirm that AM glutamatergic neurons constitute a necessary neural substrate for CFA-induced pain-anxiety comorbidity and that ablation of this neuronal population concurrently alleviates both pain hypersensitivity and anxiety-like behaviors.

### 3.7 Bidirectional chemogenetic manipulation of AM^CaMKIIα^ neuron activity reversibly modulates pain sensitivity and anxiety-like behaviors

To elucidate the causal role of AM glutamatergic neurons in pain-anxiety comorbidity, we employed chemogenetic activation and inhibition strategies to verify the sufficiency and necessity of their regulation.

**Figure 7:**
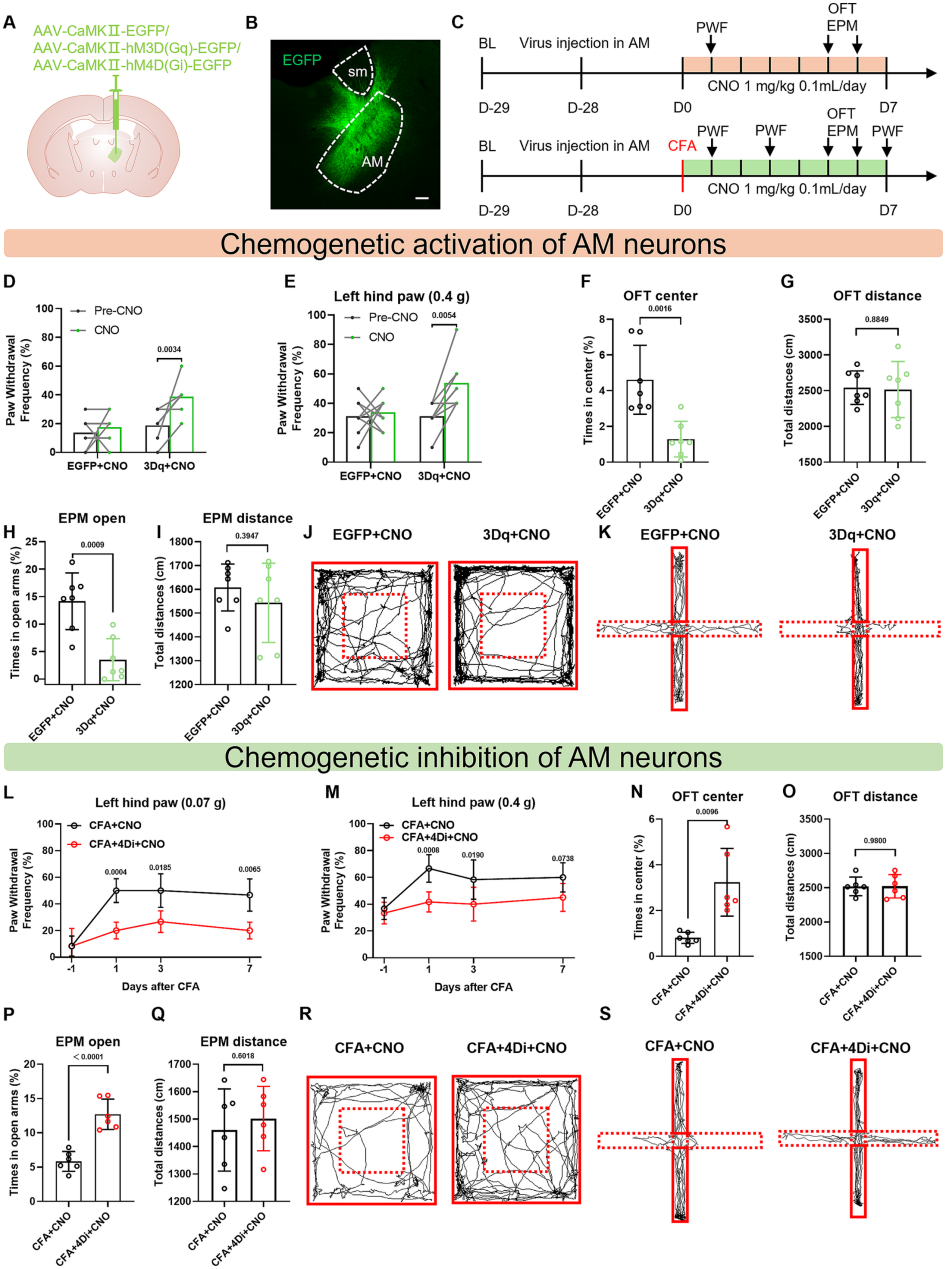
Bidirectional chemogenetic manipulation of AM^CaMKIIα^ neuron activity reversibly modulates pain sensitivity and anxiety-like behaviors. **(A)** Schematic diagram of the viral injection site. **(B)** Fluorescence image showing viral expression in the AM region. **(C)** Behavioral testing flowchart. **(D, E)** After CNO administration, the hM3Dq + CNO group showed a significantly increased mechanical paw withdrawal frequency on the left hind paw. **(F, G)** The hM3Dq + CNO group spent less time in the center zone of the open field test, with no significant difference in total distance traveled. **(H, I)** The hM3Dq + CNO group spent less time in the open arms of the elevated plus maze, with no significant difference in total distance traveled. **(J)** Representative movement trajectories of mice in the open field test. **(K)** Representative movement trajectories of mice in the elevated plus maze test. **(L, M)** Compared to the CFA + CNO group, the CFA + hM4Di + CNO group showed a significantly reduced mechanical paw withdrawal frequency on days 1, 3, and 7 post-CFA injection. **(N, O)** The CFA + hM4Di + CNO group spent more time in the center zone of the open field test, with no significant difference in total distance traveled. **(P, Q)** The CFA + hM4Di + CNO group spent more time in the open arms of the elevated plus maze, with no significant difference in total distance traveled. **(R)** Representative movement trajectories of mice in the open field test. **(S)** Representative movement trajectories of mice in the elevated plus maze test.

In naive mice, specific activation of AM glutamatergic neurons (via AAV-CaMKIIa-hM3D(Gq)-EGFP virus combined with consecutive 6-day CNO administration) independently induced pain hypersensitivity and anxiety-like behaviors (Figures 7A–C). The 3Dq + CNO group after CNO activation showed a significantly increased paw withdrawal frequency in response to mechanical stimuli (0.07 g and 0.4 g Von Frey filaments) (Figures 7D,E). Concurrently, the time spent exploring the center zone in the open field test was reduced, and the time spent in the open arms of the elevated plus maze was decreased (Figures 7F,H,J,K). These results indicate that hyperexcitation of AM glutamatergic neurons is sufficient to drive pain-anxiety comorbidity. There was no significant difference in the total distance travelled by the two groups of mice in the OFT and EPM (Figures 7G,I).

In CFA inflammatory pain model mice, inhibition of AM glutamatergic neurons (via AAV-CaMKIIa-hM4D(Gi)-EGFP virus combined with CNO) concurrently reversed pain hypersensitivity and anxiety behaviors. The inhibition group exhibited a significantly reduced paw withdrawal frequency in response to mechanical stimuli (Figures 7L,M). The time spent in the center zone of the open field was increased, and the time spent in the open arms of the elevated plus maze was elevated (Figures 7N,Q,R,S). These results confirm that the pathological activation of AM glutamatergic neurons is necessary for maintaining chronic pain-anxiety comorbidity. There was no significant difference in the total distance traveled by the two groups of mice in the OFT and EPM (Figures 7O,Q).

### 3.8 Bidirectional optogenetic manipulation of the AM-ACC circuit reversibly modulates pain sensitivity and anxiety-like behaviors

**Figure 8:**
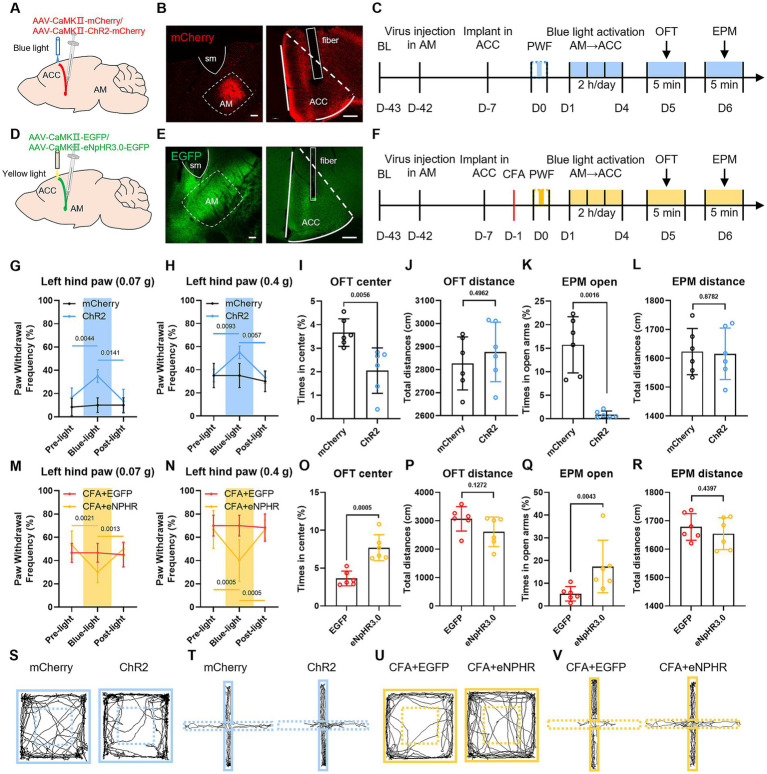
Bidirectional optogenetic manipulation reveals the spatiotemporal causal mechanism of the AM-ACC circuit in pain-anxiety comorbidity. **(A, D)** Schematic diagram of viral injection: AAV2/9-CaMKIIα-ChR2-mCherry or AAV2/9-CaMKIIα-eNpHR3.0-EGFP virus was injected into the right AM region of naive mice, and an optical fiber was implanted above the ACC to establish a closed-loop optogenetic control system. **(B, E)** Representative fluorescence images showing viral injection sites in the AM and optical fiber implantation sites in the ACC. **(C, F)** Experimental design flowchart. **(G, H)** During blue light stimulation, the ChR2 group showed a significantly increased paw withdrawal frequency in response to 0.07 g and 0.4 g Von Frey filament stimulation compared to the baseline. **(I, J)** After consecutive blue light intervention (2 h/day), the ChR2 group spent less time in the center zone of the open field test. **(K, L)** The ChR2 group showed a reduced percentage of time spent in the open arms of the elevated plus maze. **(M, N)** During yellow light inhibition, the eNpHR3.0 group showed a significantly reduced paw withdrawal frequency in response to Von Frey filament stimulation compared to the baseline. **(O, P)** After consecutive yellow light intervention (2 h/day), the eNpHR3.0 group spent more time in the center zone of the open field test. **(Q, R)** The eNpHR3.0 group showed an increased percentage of time spent in the open arms of the elevated plus maze. **(S–V)** Representative movement trajectories of mice in the open field test and elevated plus maze test during optogenetic activation and inhibition experiments.

To delineate the spatiotemporally specific function of the AM-ACC circuit and its causal role in pain-anxiety comorbidity, we employed a bidirectional optogenetic intervention strategy. In naive mice, the right AM region was stereotaxically injected with AAV-CaMKIIa-ChR2-mCherry or AAV-CaMKIIa-mCherry virus, and an optical fiber was implanted above the ACC (Figures 8A–C). Real-time blue light stimulation (473 nm, 20 Hz, 10 ms pulse width, 2 min on/2 min off) to activate AM fibers projecting to the ACC resulted in a significantly increased paw withdrawal frequency in response to 0.07 g and 0.4 g Von Frey filaments in the ChR2 group during light stimulation compared to the mCherry virus control group (Figures 8G,H). After 4 consecutive days of intervention, the ChR2 group spent a reduced percentage of time in the center zone of the open field test and a reduced percentage of time in the open arms of the elevated plus maze (Figures 8I,K,S,T). These results indicate that activation of the AM-ACC circuit can independently induce pain hypersensitivity and anxiety-like behaviors in healthy individuals, mimicking the comorbid phenotype of chronic pain. There was no significant difference in the total distance travelled by the two groups of mice in the OFT and EPM (Figures 8J,L).

In CFA inflammatory pain model mice, the right AM region was injected with AAV-CaMKIIα-eNPHR-EYFP or AAV-CaMKIIa-EGFP virus, and an optical fiber was implanted above the ACC (Figures 8D–F). Yellow light inhibition (589 nm, 20 Hz, 10 ms pulse width, 2 min on/2 min off) of AM fibers projecting to the ACC resulted in a significantly reduced paw withdrawal frequency in response to 0.07 g and 0.4 g Von Frey filaments in the CFA + eNpHR3.0 group during light inhibition compared to the CFA + EGFP group (Figures 8M,N). After 4 consecutive days of intervention, the CFA + eNpHR3.0 group spent an increased percentage of time in the center zone of the open field test (Figures 8O,U) and an increased percentage of time in the open arms of the elevated plus maze (Figures 8Q,V). This confirms that inhibiting the hyperactive AM-ACC circuit concurrently reverses CFA-induced pain hypersensitivity and anxiety behaviors. There was no significant difference in the total distance travelled by the two groups of mice in the OFT and EPM (Figures 8P,R).

In conclusion, bidirectional optogenetic manipulation of the AM-ACC circuit establishes its causal necessity: activation of this circuit can precisely induce the pain-anxiety comorbidity phenotype in healthy subjects, while its inhibition effectively alleviates comorbid symptoms in chronic pain models.

### 3.9 CRH neurons mediate the pain-anxiety comorbidity effects of the AM-ACC circuit

To elucidate the core regulatory role of ACC^CRH^ neurons within the AM-ACC circuit and its molecular mechanism, we conducted systematic validation using a combined optogenetic–chemogenetic dual intervention model.

**Figure 9:**
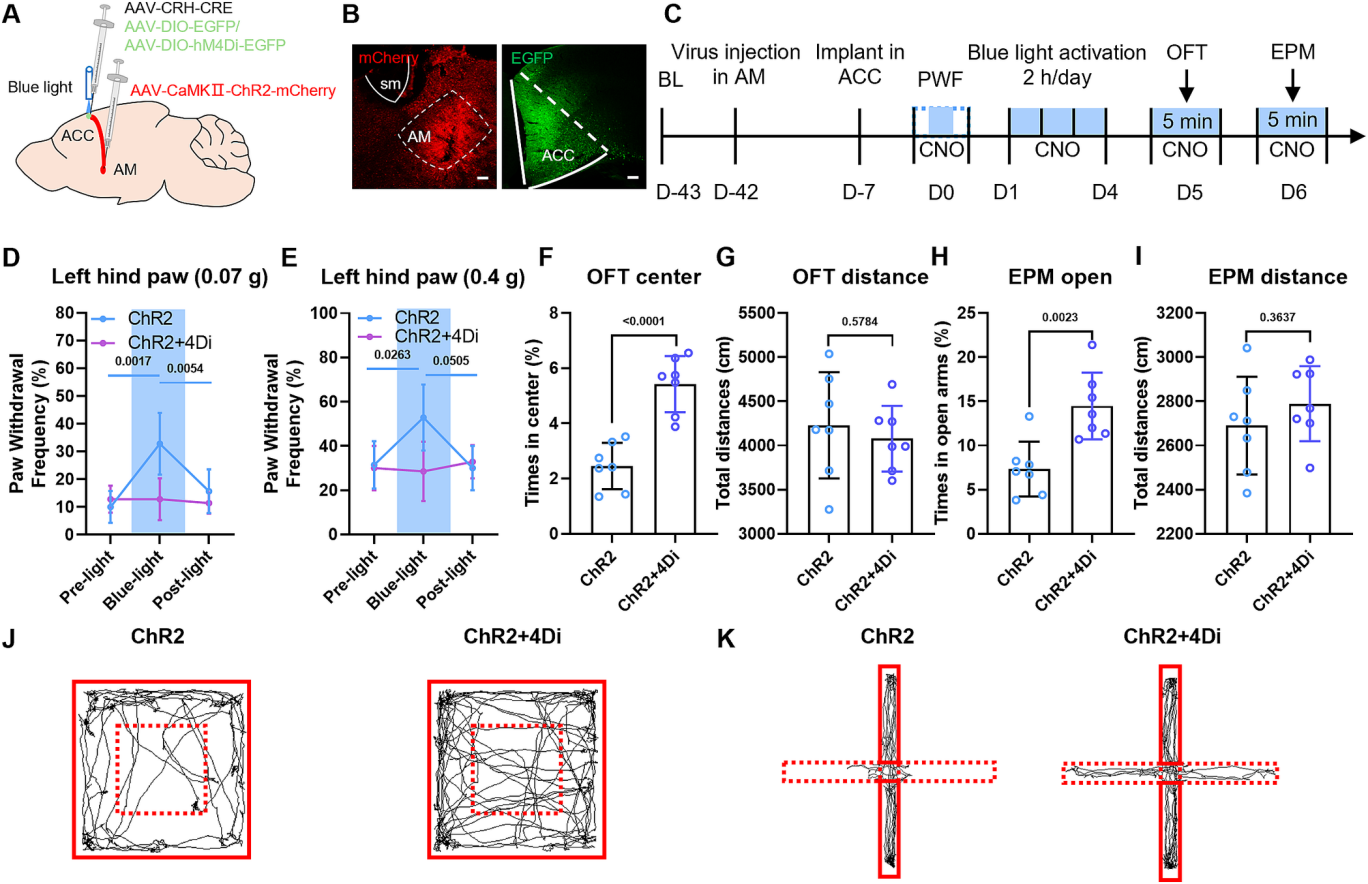
Precise dissection of CRH neuron-mediated pain-anxiety comorbidity effects in the AM-ACC circuit. **(A)** Schematic diagram of the combined optogenetic–chemogenetic dual intervention viral injection strategy: AAV2/9-CaMKIIα-ChR2-mCherry was injected into the AM region to activate the circuit, while AAV2/9-CRH-hM4D(Gi)-EGFP was co-injected into the ACC to inhibit CRH neurons. **(B)** Representative fluorescence images showing viral injection sites in the AM and ACC. **(C)** Experimental design flowchart. **(D, E)** The dual intervention group (ChR2 + hM4Di) showed a significantly reduced mechanical pain response frequency compared to the single intervention group (ChR2). **(F, G)** The combined optogenetic–chemogenetic dual intervention group exhibited restored time spent in the center zone of the open field test. **(H, I)** The ChR2 + hM4Di group showed an increased percentage of time spent in the open arms. **(J, K)** Representative movement trajectories of mice from the anxiety-like behavior tests in the combined optogenetic–chemogenetic dual intervention experiment.

In the combined optogenetic–chemogenetic dual intervention model, AM fibers projecting to the ACC were activated by injecting AAV-CaMKIIa-ChR2-mCherry into the AM region, while co-injecting AAV-CRH-Cre and AAV-DIO-hM4D(Gi)-EGFP or AAV-DIO-EGFP (Figures 9A–C). Behavioral results showed that compared to the only optogenetic AM-ACC activation group (ChR2), the dual intervention group (ChR2 + hM4Di) exhibited a significantly reduced paw withdrawal frequency in response to mechanical stimulation during 473 nm blue light stimulation (Figures 9D,E). Further anxiety behavior tests demonstrated that inhibition of CRH neurons partially reversed the circuit activation effects: the time spent in the center zone of the open field test increased (Figures 9F,J), and the percentage of time spent in the open arms of the elevated plus maze increased (Figures 9H,K). There was no significant difference in the total distance travelled by the two groups of mice in the OFT and EPM (Figures 9G,I).

In conclusion, CRH neurons in the ACC serve as the core hub through which the AM-ACC circuit drives pain-anxiety comorbidity: inhibiting their activity can significantly block the transmission of pathological signals from AM inputs, with the intervention effects manifesting simultaneously in both sensory (mechanical allodynia) and emotional (anxiety behaviors) dimensions.

## 4 Discussion

The comorbidity mechanism of chronic pain and anxiety constitutes a pivotal challenge in clinical neuroscience. The present study reveals that the AM^CaMKIIα^-ACC^CRH^ pathway acts as a specific neural circuit driving pain-anxiety comorbidity. In the CFA-induced chronic inflammatory pain model, glutamatergic neurons in the AM exhibited pathological hyperactivation, and their glutamatergic projections activated CRH neurons in the ACC, thereby inducing mechanical hyperalgesia and anxiety-like behaviors. Activation of AM^CaMKIIα^ neurons, ACC^CRH^ neurons or the AM^CaMKIIα^-ACC neural circuit was sufficient to elicit hyperalgesia and anxiety-like behaviors in naive mice; in contrast, inhibition of these neurons or the aforementioned circuit reversed the pain-anxiety comorbid phenotypes in CFA model mice, which demonstrated that this pathway fulfills the causal correlation of sufficiency and necessity for mediating the comorbidity.

### 4.1 ACC^CRH^ neurons mediate chronic inflammatory pain and anxiety-like behaviors

CRH neurons are densely distributed mainly in the paraventricular nucleus of the hypothalamus (PVN) and regulate the stress endocrine response via the HPA axis. Recent studies have found that CRH neurons are also widely expressed in the cerebral cortex and limbic system, whose functions go beyond the traditional endocrine scope and have gradually evolved into key hubs for neural circuit regulation. By integrating excitatory and inhibitory signals from specific brain regions, CRH neurons finely regulate anxiety behaviors in a bidirectional manner. For instance, CRH neurons in brain regions such as the amygdala and mPFC are involved in chronic pain and anxiety-like behaviors (Mazzitelli et al., 2022; Tsushima et al., 2021; Hein et al., 2021; Yu et al., 2024). Inhibition of amygdalar CRH neurons can alleviate chronic pain and anxiety-like behaviors (Mazzitelli et al., 2022; Tsushima et al., 2021; Hein et al., 2021). CRH neurons in the mPFC receive glutamatergic excitatory projections from the ventral hippocampal vCA1, and induce anxiety/depression-like behaviors by inhibiting the excitability of mPFC pyramidal neurons under conditions of chronic pain and stress (Lv et al., 2024). In contrast, CRH neurons in the NAc project to the BNST, and promote arousal and alleviate anxiety by releasing GABA to inhibit the activity of local neurons (Pan et al., 2024). Previous studies have indicated that the ACC is a key brain region involved in the regulation of chronic pain and anxiety, and CRH is also widely expressed in the ACC (Zhang et al., 2019; Yoshida et al., 2018). However, whether ACC^CRH^ neurons are involved in the regulation of chronic pain and anxiety remains unclear. Our study results demonstrated that chemogenetic activation of ACC^CRH^ neurons induced pain and anxiety-like behaviors in mice, while chemogenetic inhibition of ACC^CRH^ neurons significantly alleviated pain and anxiety-like behaviors in CFA model mice, confirming that ACC^CRH^ neurons are involved in the regulation of pain and anxiety-like behaviors in mice.

However, since CRH neurons colocalize with other neurotransmitters, and the properties of colocalized neurotransmitters vary significantly across different brain regions, the input function of CRH neurons to downstream neurons is also regulated by the type of colocalized neurotransmitters and the phenomenon of neurotransmitter co-release. For example, CRH neurons in the PVN and subthalamic nucleus (STN) mainly co-express glutamatergic markers (Tseng et al., 2022), whereas CRH neurons in the lateral hypothalamus (LH)(Li et al., 2023), mPFC (Lv et al., 2024) and NAc (Pan et al., 2024) are often colocalized with GABAergic markers. If glutamate co-release occurs in these neurons, the two transmitters can jointly drive excitatory output (manifested as depolarization). If GABA co-release occurs, it activates postsynaptic Cl^−^ channels, which may antagonize the excitatory effect of CRH. This co-release mechanism significantly enhances the precision of neural regulation. The neurotransmitter properties of CRH neurons in the ACC have not been fully elucidated, and several studies have suggested that these neurons may colocalize with both glutamatergic and GABAergic markers. Therefore, the neurotransmitter type of ACC^CRH^ neurons and the specific mechanism of its co-release effect require further investigation.

The present study mainly elucidates the upstream regulatory mechanism by which glutamatergic inputs from the AM activate ACC^CRH^ neurons, confirming this circuit as a core pathway driving chronic inflammatory pain-anxiety comorbidity. The functional output of the ACC in mediating pain modulation is also dependent on its downstream projection pathways: the ACC not only sends projections to descending antinociceptive nuclei such as the periaqueductal gray (PAG) and rostral ventromedial medulla (RVM) to modulate pain (Hohenschurz-Schmidt et al., 2020), but also projects directly to the spinal cord to facilitate spinal processing of nociceptive signals (Chen et al., 2018). In summary, in all likelihood, ACC^CRH^ neurons also need to directly or indirectly regulate spinal neurons to mediate the descending facilitation of pain, thereby contributing to the maintenance of mechanical hyperalgesia. This may represent a key downstream mechanism underlying the mediation of pain-anxiety comorbidity by the AM^CaMKIIα^-ACC^CRH^ circuit, a hypothesis that has not been verified in the present study. Subsequent research can employ neural circuit tracing, optogenetic/chemogenetic manipulation and other techniques to verify the structural connectivity and functional effects of the ACC^CRH^ downstream circuits, so as to refine the central regulatory network underlying chronic inflammatory pain-anxiety comorbidity.

### 4.2 AM^CaMKIIα^ neurons mediate chronic inflammatory pain and anxiety-like behaviors

Since James Papez proposed the closed-loop circuit of “hippocampus-mammillary body-anterior thalamic nucleus-cingulate gyrus” as the neural substrate of emotion in 1937(Papez, 1995), the academic community’s understanding of its functions has undergone a tortuous yet profound evolution. Recent studies have indicated that damage to the key nodes of this circuit can lead to severe episodic memory impairment (Ji et al., 2020; Jang and Kwon, 2018; Yang et al., 2016), and its functional localization has thus been revised to a memory system, which challenges the original “emotional hypothesis.” As a key hub of the Papez circuit, the AM has been the focus of intensive research in recent years for its role in the encoding and consolidation of emotional memory (Aggleton et al., 2022; Kamali et al., 2023). However, several studies have shown that the AM is also involved in the regulation of pruritus (Deng et al., 2020), and AM neurons exhibit excitatory responses when the body is subjected to noxious stimuli (Dostrovsky and Guilbaud, 1990), suggesting that it may participate in the transmission and regulation of sensory information. In our study, results demonstrated that inhibition of AM^CaMKIIα^ neurons in mice significantly alleviated the pain and anxiety-like behaviors induced by the CFA model, which re-emphasizes the functional localization of the AM in negative emotion (anxiety) and pain. As a component of the thalamus, it is deeply involved in the processing of pain sensory signals and the induction of anxiety emotion.

### 4.3 The AM^CaMKIIα^-ACC neural circuit mediates chronic inflammatory pain and anxiety-like behaviors

Our study revealed that activation of the AM-ACC neural circuit, a key node of the Papez circuit, directly induces pain and anxiety-like behaviors. Together with evidence of white matter abnormalities in the Papez circuit of patients with anxiety disorders (Li et al., 2023), our study has reaffirmed the critical causal role of the Papez circuit in negative emotions, particularly anxiety. This spiral cognitive journey—evolving from the proposal of its emotional function (1937) to the recognition of its dominant role in memory, and then to the reaffirmation of its function in emotions (especially anxiety)—not only highlights the challenges in understanding the complex neural circuits of the brain, but also ultimately reveals that the Papez circuit is actually a dynamic network integrating mnemonic and emotional functions.

Another study has reported that the AM-ACC circuit mediates histaminergic itch-associated scratching behavior (Deng et al., 2020), which forms a highly meaningful association with the finding of the present study that this circuit is involved in chronic pain-anxiety comorbidity. Regarding the relationship between itch and pain, the academic community initially proposed that itch represents a low-intensity form of pain perception, meaning a stimulus would elicit itch when its intensity fails to reach the pain threshold (Andrew and Craig, 2001; Handwerker and Schmelz, 2009). However, with the advancement of research, it has now been clearly established that the two are not merely distinguished by stimulus intensity. Instead, while sharing certain phenotypic similarities and common regulatory mechanisms (Gunawardena et al., 2023; Ji et al., 2019; Moore et al., 2018), they exhibit fundamental differences—they possess distinct types of peripheral primary sensory receptors and are activated by different specific stimuli, which further leads to numerous divergences in their central signal transduction processes (Liu and Ji, 2013). The ACC, a higher brain center, serves as a core integrative hub for somatosensory information and associated emotional signals and plays a pivotal role in the central regulation of both pain (Bliss et al., 2016) and itch (Zhang et al., 2025). This provides an important structural basis for the AM-ACC circuit to independently modulate itch and chronic pain-anxiety comorbidity, and thus the functional findings of the two studies regarding this circuit are not contradictory. Furthermore, the present study did not conduct a generalized investigation into the AM-ACC circuit’s overall regulation of the ACC; instead, we focused on the specific corticotropin-releasing hormone (CRH) neurons within the ACC. The specificity of this target is one of the key reasons for the functional divergence of the AM-ACC circuit, whereby it mediates pain-anxiety comorbidity in our study and modulates itch in the other study. In addition, it is noteworthy that increased grooming duration is one of the typical manifestations of anxiety-like behaviors in mice, and this behavior shows a striking phenotypic similarity to the scratching behavior observed in histaminergic itch studies. This also reflects the close connection between somatosensory regulation and emotional regulation at the behavioral level.

The present study exclusively used male mice for all investigations, and its conclusions should therefore be cautiously extrapolated to female individuals. Previous studies have demonstrated that there are no significant differences in the anatomical distribution of CRH neurons and basal systemic CRH levels between male and female mice, whereas sex-specific divergence exists in CRH release levels and neuronal excitability under different conditions. CRH neurons in males highly express androgen receptors (AR); under stress conditions, androgens directly enhance neuronal excitability by activating AR, which in turn causes hyperactivation of the HPA axis. This leads to a substantial elevation in the levels of CRH, adrenocorticotropic hormone and glucocorticoids, triggering more severe immunosuppressive effects (e.g., thymic atrophy and increased thymocyte apoptosis)(Meng et al., 2025), which confirms that AR is the core molecular mechanism underlying the high stress sensitivity in males. In contrast, females lack the AR signaling pathway but exhibit a lower stress-induced threshold: mild stimuli such as exposure to a novel environment can trigger short-term synaptic potentiation (STP), while social interaction can significantly buffer such neuroplastic changes (Sterley et al., 2018). Collectively, males are more prone to developing stable and persistent neural effects following stress exposure.

In conclusion, our study demonstrated that the neural circuit from AM^CaMKIIα^ neurons to ACC^CRH^ neurons mediates chronic inflammatory pain and anxiety-like behaviors. This study opens up new perspectives for the functional investigation of the anterior thalamic nucleus and also provides novel therapeutic targets for the treatment of chronic pain.

## 5 Conclusion

This study identifies the AM^CaMKIIα^-ACC^CRH^ neural circuit as a pivotal driver of chronic inflammatory pain and anxiety comorbidity. Activation of AM^CaMKIIα^ neurons, ACCCRH neurons, or the AM-ACC circuit induces pain hypersensitivity and anxiety-like behaviors in naive mice, while their inhibition reverses these comorbid phenotypes in CFA-induced pain models. Mechanistically, AM^CaMKIIα^ neurons innervate and enhance the excitability of ACC^CRH^ neurons via glutamatergic projections, forming a functional circuit that integrates pain sensory and emotional signals. These findings enrich our understanding of pain-emotion integration mechanisms, expand the functional connotation of the Papez circuit, and provide a novel specific target for the clinical treatment of chronic pain combined with anxiety.

## Data Availability

The original contributions presented in the study are included in the article/Supplementary material, further inquiries can be directed to the corresponding authors.
