# Supplementary material for: CRH neurons in the AM-ACC circuit drive chronic pain and anxiety comorbidity
Source: Front Neurosci. 2026 Apr 10;20:1809769. doi: 10.3389/fnins.2026.1809769 (PMC13106490; doi:10.3389/fnins.2026.1809769)
Supplement: Supplementary file 1 [file Data_Sheet_1.pdf]

Supplementary Material

1 Supplementary Figures and Tables

1.1 Supplementary Figures

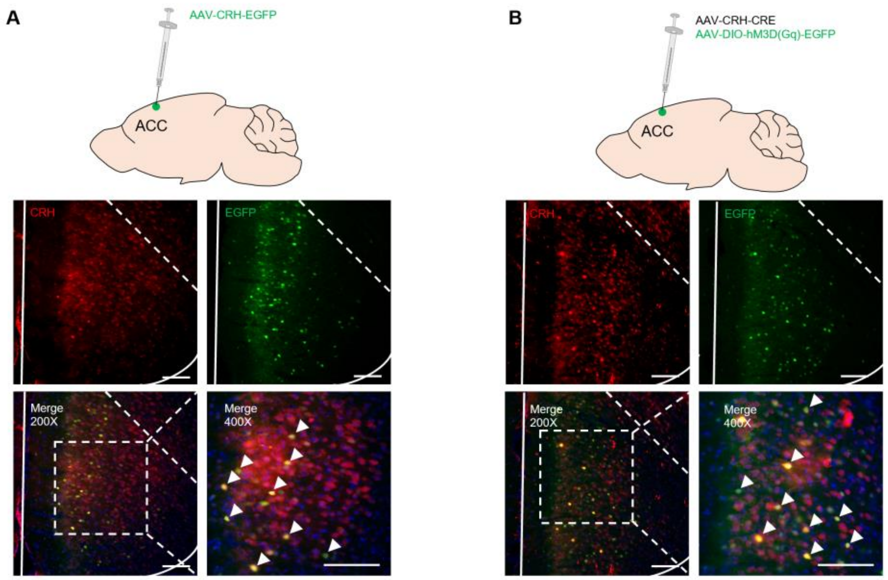

Supplementary Figure 1. The CRH promoter virus exhibits neuron-specificity. (A) The green fluorescence of AAV-CRH-EGFP virus was highly colocalized with the red CRH signal detected by immunoflu-orescence staining. (B) The green fluorescence of AAV-CRH-CRE and AAV-DIO-hM3D(Gq)-EGFP viruses was highly colocalized with the red CRH signal detected by immunofluorescence staining.
